# Patient-Derived Orthotopic Xenograft (PDOX) Models of Melanoma †

**DOI:** 10.3390/ijms18091875

**Published:** 2017-08-31

**Authors:** Robert M. Hoffman

**Affiliations:** 1 AntiCancer Inc., 7917 Ostrow Street, San Diego, CA 92111, USA; all@anticancer.com; Tel.: +1-858-654-2555; Fax:+1-858-268-4175; 2 Department of Surgery, University of California, San Diego, CA 92103-8220, USA

**Keywords:** melanoma, patient-derived orthotopic xenografts, PDOX, targeted therapy, personalized therapy

## Abstract

Metastatic melanoma is a recalcitrant tumor. Although “targeted” and immune therapies have been highly touted, only relatively few patients have had durable responses. To overcome this problem, our laboratory has established the melanoma patient-derived orthotopic xenograft (PDOX) model with the use of surgical orthotopic implantation (SOI). Promising results have been obtained with regard to identifying effective approved agents and experimental therapeutics, as well as combinations of the two using the melanoma PDOX model.

## 1. Introduction

Metastatic melanoma has a survival rate of 7–29%, depending on the site of metastasis [1]. Recent use of targeted chemotherapy and immunotherapy has not significantly increased the survival rate [2]. The standard first-line therapy has been decarbazine and cisplatinum (CDDP), with limited efficacy [3,4,5]. Vemurafenib (VEM) has had some success as a targeted therapy of melanoma that has the BRAF-V600E mutation [3,6,7,8,9]. 

PD-1/PD-L1 immunotherapy has shown promise with melanoma, but is limited by tumor infiltration of activated T cells [5], and has not increased the survival rate [2]. 

Stage III and IV melanoma is almost never curable, due to a lack of effective drugs, resistance to immunotherapy and tumor heterogeneity [10]. Chemotherapy and radiotherapy of melanoma are also limited by melanin [11]. Individualized and precision therapy is needed for melanoma. 

To achieve this goal, the patient-derived orthotopic xenograft (PDOX) nude mouse model using surgical orthotopic implantation (SOI) [12] has been developed in our laboratory. PDOX models of pancreatic [13,14,15,16], breast [17], ovarian [18], lung [19], cervical [20], colon [21,22,23], stomach [24] and sarcoma cancers [25,26,27,28,29] have been developed. Fluorescence-guided surgery [14,23,30] and tumor-targeting bacteria [15,27,28,29,31] have been developed with the PDOX models. The tumor microenvironment has also been studied in the PDOX models [32,33]. The PDOX models have been shown to have advantages over subcutaneous-transplant models, particularly with metastasis [12].

The present report reviews our laboratory’s experience with PDOX models of melanoma, and the ability of the PDOX models to identify effective currently-used—as well as experimental—therapeutics.

Tumor-targeting *Salmonella typhimurium* A1-R (*S. typhimurium* A1-R) contains auxotrophic mutations for leucine (leu) and arginine (arg), and therefore does not continuously infect normal tissue [34,35]. *S. typhimurium* A1-R has shown significant efficacy against mouse models of various cancer cell lines including prostate [36,37], breast [38,39,40], lung [41,42], pancreatic [15,31,43,44,45], ovarian [46,47] stomach [48], and cervical cancers [49], as well as sarcoma cell lines [50,51,52], glioma [53,54], and the PDOX models mentioned above [15,27,28,29,31].

## 2. Results and Discussion

### 2.1. Patient-Derived Melanoma Growing Orthotopically in Nude Mice

**Figure 1:**
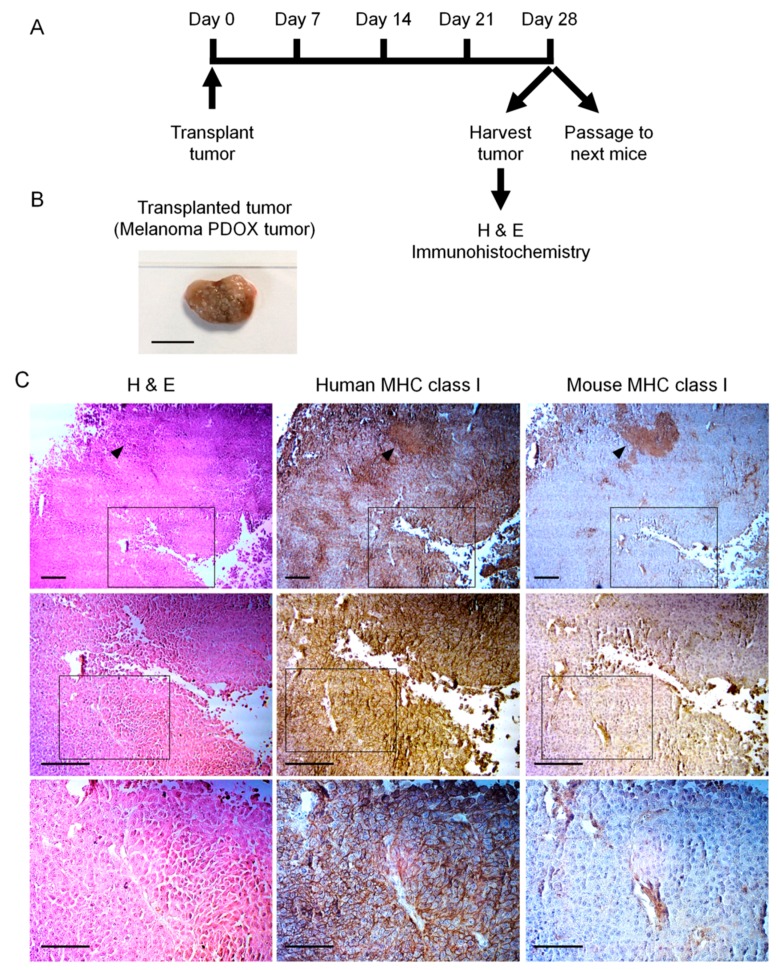
(**A**) Experimental scheme; (**B**) Patient-derived orthotopic xenograft (PDOX) melanoma after 28 days of growth, Scale bar: 10 mm; (**C**) hematoxylin and eosin- (H&E)-stained tumor sections (left column), human MHC class I (HLA; middle column) immunohistochemistry, mouse MHC class I (right column), mouse MHC immunohistochemistry. The human cancer cells expressed human MHC class I and the mouse stromal cells and blood vessels expressed mouse MHC. Magnified views of boxed region in the upper rows are indicated at the middle rows and magnified views of boxed region in the middle rows are indicated in the lower rows. Scale bars: 200 μm (top and middle row), 100 μm (bottom row) [34].

Our initial experience with a melanoma PDOX was with a tumor obtained from the University of California San Diego (UCSD), which was subdermally transplanted orthotopically [34]. The melanoma PDOX grew and expressed human MHC class I protein. In contrast, the tumor microenvironment only reacted with the mouse MHC class I antibody. Thus, the growing PDOX tumor was of human origin (Figure 1) [34].

### 2.2. S. typhimurium A1-R Was Highly Effective on the Patient-Derived Orthotopic Xenograft (PDOX) Melanoma in Nude Mice

**Figure 2:**
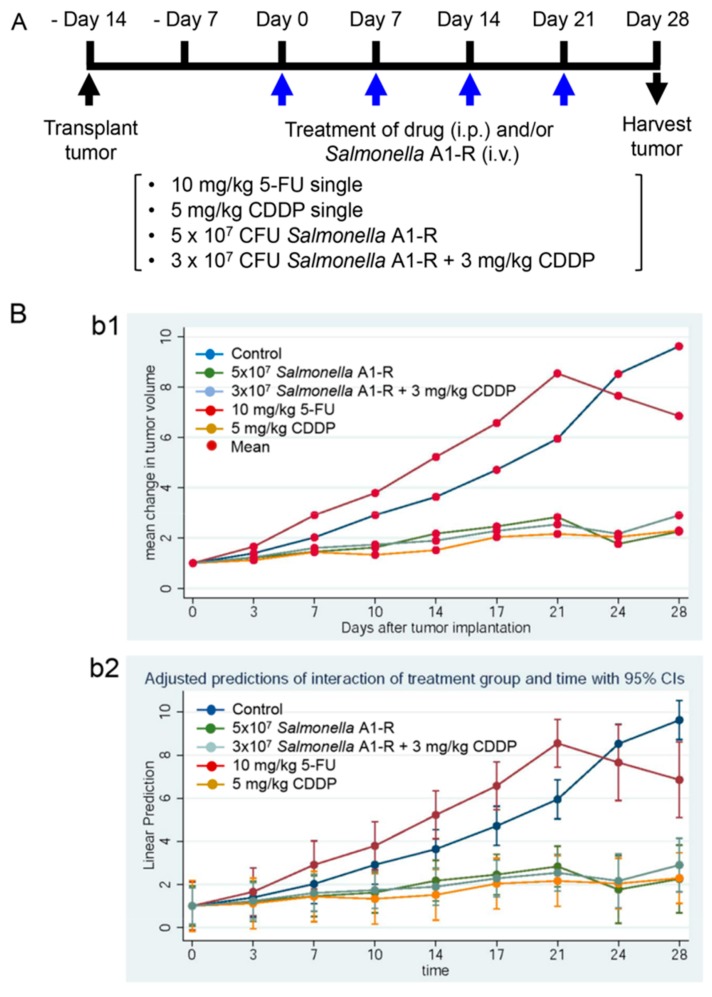

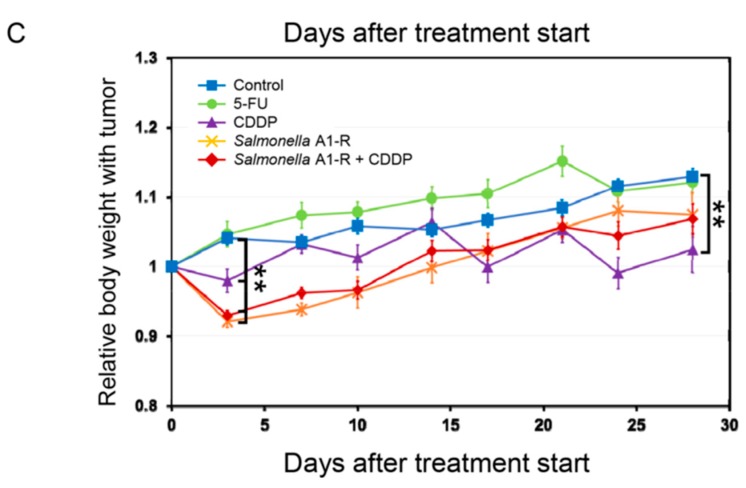
Efficacy of *S. typhimurium* A1-R, 5-fluorouracil (5-FU) and cisplatinum (CDDP) on a melanoma PDOX model. (**A**) Experimental scheme; (**b1**) mean change in tumor volume plotted against time, as shown in untreated and control tumors; (**b2**) linear prediction versus time curves for untreated control and treated tumors; (**C**) body weight comparison in nude mice after *S. typhimurium* A1-R and/or 5-FU and CDDP therapy [34]. ** *p* < 0.01, compared with the untreated control group.

*S. typhimurium* A1-R, expressing green fluorescent protein (GFP), extensively targeted the tumor, with very few GFP-expressing bacteria found in other organs (i.e., demonstrating high tumor selectivity). *S. typhimurium* A1-R strongly inhibited the growth of the melanoma (Figure 1). *S. typhimurium* A1-R, cisplatinum (CDDP), and a combination of *S. typhimurium* A1-R and CDDP, were all highly effective on the melanoma PDOX (Figure 2) [34].

### 2.3. PDOX Model of a BRAF-V600E Mutant Melanoma

**Figure 3:**
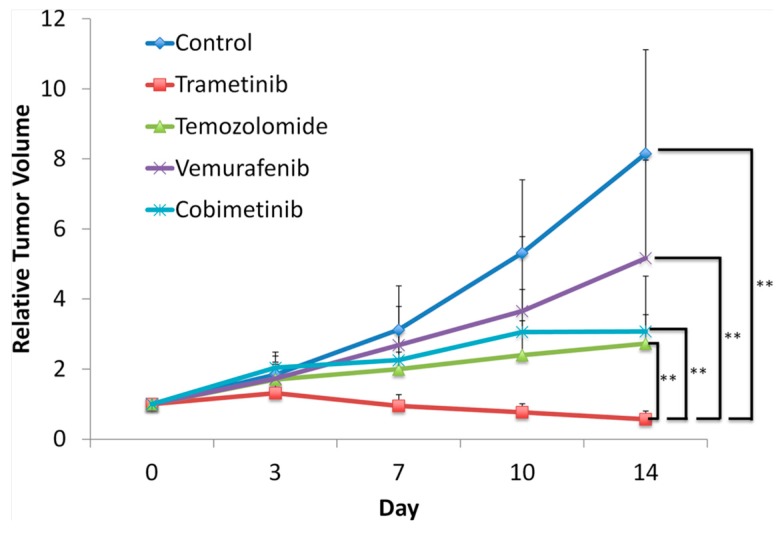
Efficacy of targeted therapies against a BRAF-V600E mutant melanoma PDOX. Relative tumor volume is the ratio of the tumor volume at any time point relative to the initial tumor volume. Only trametinib (TRA) could regress the tumor. Vemurafenib (VEM) was not very effective despite the fact that it targets the BRAF-V600E mutation in this tumor. ** *p* ≤ 0.0001. Error bars = SD [55].

A BRAF-V600E mutant melanoma PDOX was established. VEM, temozolomide (TEM), trametinib (TRA) and cobimetinib (COB) were all effective against it. TRA treatment caused tumor regression (Figure 3). The PDOX was expected to be sensitive to VEM, since VEM targets the BRAF-V600E mutation. However, in this case, TRA was much more effective than VEM [55]. This result shows that the BRAF-V600E mutation is probably not a major factor in promoting this melanoma, and that genomic profiling by itself is insufficient to direct therapy. 

**Figure 4:**
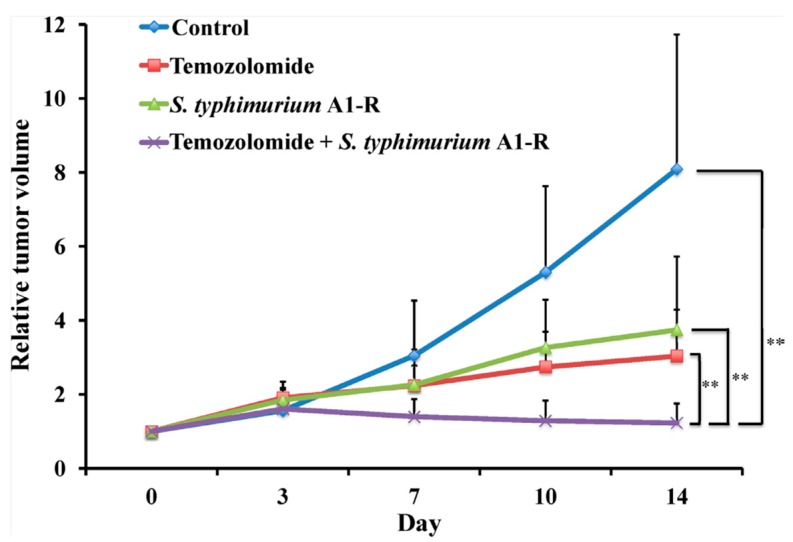
BRAF-V600E mutant melanoma PDOX. Tumor size of the untreated control mice increased over time. Tumors treated with TEM or *S. typhimurium* A1-R were inhibited. Tumors treated with TEM combined with *S. typhimurium* A1-R regressed. ** *p* < 0.01. Error bars = SD [56].

In a subsequent study with this BRAF-V600E mutant melanoma PDOX, TEM combined with *S. typhimurium* A1-R was significantly more effective than either *S. typhimurium* A1-R and TEM alone, causing regression of the tumor (Figure 4). Confocal microscopy showed that the *S. typhimurium* A1-R could directly target the melanoma PDOX and cause tumor necrosis [56]. 

**Figure 5:**
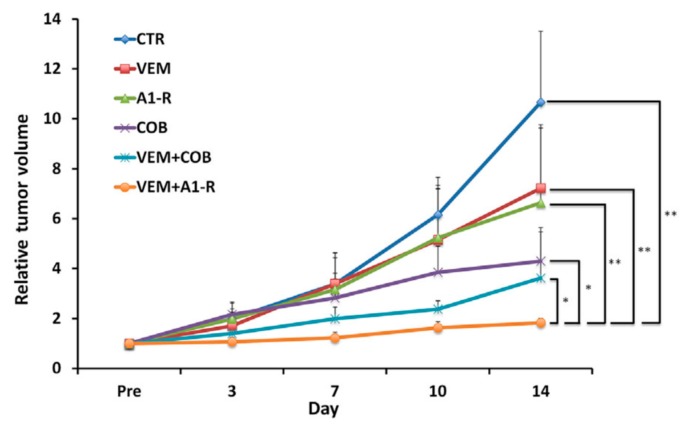
Tumor growth curves of the treated and untreated BRAF-V600E mutant melanoma PDOX. Line graph shows tumor volume at each point relative to the initial tumor volume. Please see Materials and Methods section for drug dose, route and schedule. ** *p* < 0.01, * *p* < 0.05. Error bars = SD [9].

In a subsequent study, VEM, *S. typhimurium* A1-R, COB, VEM combined with COB, and VEM combined with *S. typhimurium* A1-R were all effective against the BRAF-V600E mutant melanoma PDOX, compared to the untreated control. VEM combined with *S. typhimurium* A1-R was the most effective compared to other therapies (Figure 5). Tumor necrosis was more extensive in the group treated with VEM combined with *S. typhimurium* A1-R [9].

**Figure 6:**
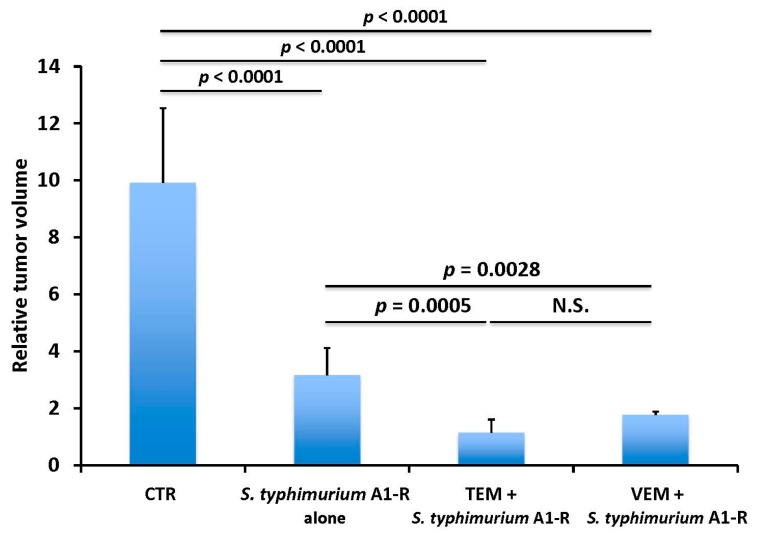
Relative tumor volume in the various treatment groups of the BRAF-V600E mutant melanoma PDOX. Bar graph shows tumor volume at post-treatment point relative to the initial pre-treatment tumor volume. Error bars = SD [57]. N.S. = not significant.

**Figure 7:**
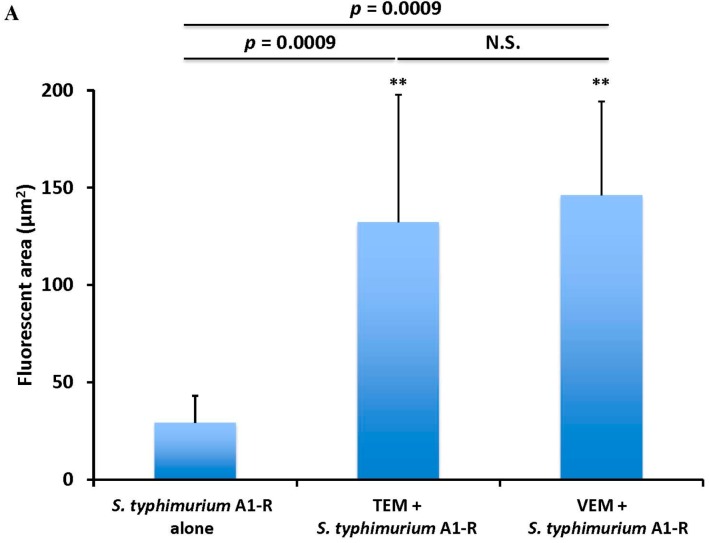

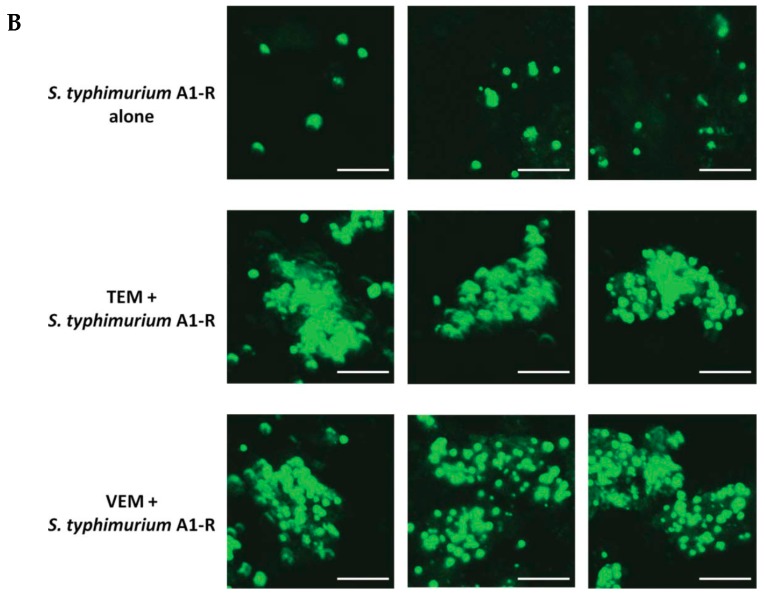
(**A**) Quantitative tumor targeting by *S. typhimurium* A1-R-GFP alone and in combination with chemotherapy on the BRAF-V600E mutant melanoma PDOX model. Bar graph shows *S. typhimurium* A1-R-GFP fluorescent area (mm^2^) for each treatment group. N.S. = not significant. Error bars = SD [57]; (**B**) fluorescence imaging of *S. typhimurium* A1-R-GFP targeting alone and in combination with chemotherapy in the melanoma PDOX. Confocal imaging with the FV1000. Scale bars: 12.5 μm.

In another study, TEM combined with *S. typhimurium* A1-R, and VEM combined with *S. typhimurium* A1-R, were significantly more effective than *S. typhimurium* A1-R alone on the BRAF-V600E mutant melanoma PDOX (Figure 6). Both VEM and TEM significantly increased the tumor targeting of *S. typhimurium* A1-R, compared to *S. typhimurium* A1-R alone, as observed by high-resolution confocal microscopy (Figure 7A,B). These results suggested that *S. typhimurium* A1-R increases the efficacy of chemotherapy, and chemotherapy increases the tumor targeting of *S. typhimurium* A1-R in the melanoma PDOX model [57].

Methionine dependence is a general metabolic defect in cancer. It has been demonstrated that methionine starvation induces a tumor-selective S/G_2_-phase cell-cycle arrest of tumor cells [58,59,60,61]. Methionine dependence is due to the excess use of methionine in aberrant transmethylation reactions, termed the Hoffman effect, and is analogous to the Warburg effect for glucose in cancer [62,63,64,65,66,67]. The excessive and aberrant use of methionine in cancer is strongly observed in [^11^C]–methionine PET imaging, where the high uptake of [^11^C]–methionine results in a very strong and selective tumor signal compared with normal tissue background. [^11^C]–methionine is superior to [^18^C]–fluorodeoxyglucose (FDG) for PET imaging, suggesting methionine dependence is more tumor-specific than glucose dependence [68,69]. A purified methionine-cleaving enzyme, methioninase (METase), from *Pseudomonas putida*, has been found previously to be an effective antitumor agent in vitro as well as in vivo [70,71,72,73]. For the large-scale production of METase, the gene from *P. putida* has been cloned in *Escherichia coli* and a purification protocol for recombinant methioninase (rMETase) has been established with high purity and low endotoxin release [74,75,76,77]. 

**Figure 8:**
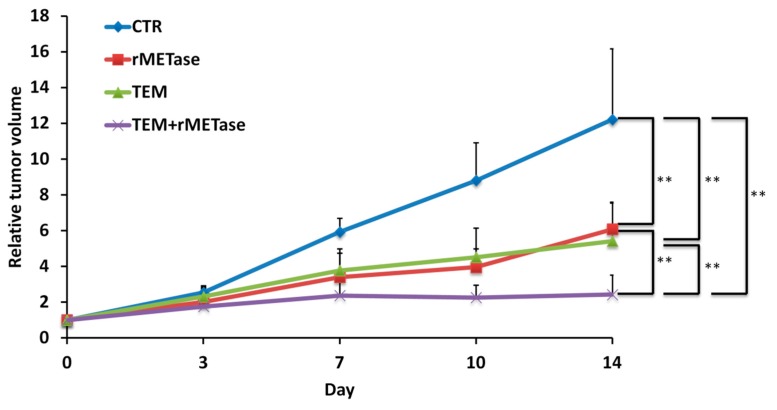
Time-coursed treatment efficacy on the BRAF-V600E mutant melanoma PDOX. Line graph shows tumor volume at each point relative to the initial tumor volume. All treatments significantly inhibited tumor growth compared to the untreated control (TEM: *p* = 0.0081, recombinant methioninase (rMETase): *p* = 0.0037, TEM/rMETase: *p* = 0.0024). In addition, TEM and rMETase combination therapy was significantly stronger than both TEM (*p* = 0.0051) and rMETase (*p* = 0.0051) alone at day 14. There was no significant difference between TEM and rMETase. ** *p* < 0.01. Error bars = SD [77].

The combination therapy of TEM and rMETase had significantly better efficacy than either therapy alone on the BRAF-V600E mutant melanoma PDOX (Figure 8). Post-treatment L-methionine levels in tumors treated with rMETase alone, or along with TEM, were significantly decreased compared to untreated controls (data not shown). These results showed that this melanoma is methionine dependent, and rMETase thereby suppresses the melanoma PDOX [77].

This review indicates that the melanoma PDOX is a promising—although still-developing—technology, able to identify effective therapy for patients, both approved and experimental. Future studies will investigate further advantages of the melanoma PDOX model. Please see references [78,79] for reviews of melanoma PDX models. Future studies will address molecular changes in the treated melanoma PDOX models described in the present report. 

## 3. Materials and Methods

### 3.1. Mice

Athymic (*nu*/*nu*) nude mice (AntiCancer Inc., San Diego, CA, USA) were used in these studies in accordance with the National Institutes of Health Guide for the Care and Use of Laboratory Animals under Assurance Number A3873-1. Animals were anesthetized with a ketamine mixture via subcutaneous injection of a 0.02 mL solution of 20 mg/kg ketamine, 15.2 mg/kg xylazine and 0.48 mg/kg acepromazine maleate for all surgeries [9,55,56,57,77]. 

### 3.2. Patient-Derived Tumors

The PDOX models from the University of California Los Angeles (UCLA) were established from a 75-year-old female patient with a melanoma of the right chest wall. The melanoma had a BRAF-V600E mutation. Tumor resection was performed in the Department of Surgery, UCLA. The tumor was provided for PDOX establishment after written informed consent was provided by the patient, and after approval was granted by the Institutional Review Board (IRB) [55]. Another patient melanoma was obtained from a patient at UCSD under IRB approval and informed patient consent [34].

### 3.3. Establishment of PDOX Models of Melanoma by Surgical Orthotopic Implantation (SOI)

Resected melanoma tissue was immediately transported to AntiCancer Inc. on ice. The BRAF-V600E mutant melanoma tumor fragments (3 mm^3^) were transplanted to the chest wall of nude mice to mimic the site from which they were resected from the patient [9,55,56,57,77]. The melanoma from UCSD was directly implanted subdermally and passaged in the back skin of nude mice [34]. All surgeries were performed under ketamine anesthesia.

### 3.4. Preparation and Administration of S. typhimurium A1-R

*S. typhimurium* A1-R (AntiCancer Inc.), expressing GFP, was cultured in LB medium (Fisher Sci., Hanover Park, IL, USA) and harvested at the late-log phase. The bacteria were washed and diluted with PBS. *S. typhimurium* A1-R was injected intravenously. A total of 5 × 10^7^ colony-forming units (CFU) of *S. typhimurium* A1-R in 100 μL phosphate-buffered saline (PBS) was administered to each mouse [36,37,38,56]. 

### 3.5. Recombinant Methionase (rMETase) Production

Recombinant l-methionine α-deamino-γ-mercaptomethane lyase (recombinant methioninase (rMETase)) (EC 4.4.1.11) from *Pseudomonas putida* was previously cloned and produced in *Escherichia coli* using previously published procedures [74].

### 3.6. Tumor Histology

The original tumor tissue and PDOX tumor tissue were fixed in 10% formalin. The fixed tumors were embedded in paraffin and then sectioned and stained. Standard bright-light microscopy was used for histopathological analysis [55].

### 3.7. Confocal Microscopy

The FV1000 confocal microscope (Olympus, Tokyo, Japan) was used for high-resolution imaging of *S. typhimurium* A1-R. Fluorescence images were obtained using the 20×/0.50 UPLAN FLN and 40×/1.3 Oil Olympus UPLAN FLN objectives [80]. 

### 3.8. Treatment Study Design in the PDOX Model of Melanoma

BRAF-V600E mutant melanoma PDOX mouse models were randomized into six groups of 10 mice each: untreated control (*n* = 10); VEM (30 mg/kg, oral (po) per week (qd) × 14); COB (5 mg/kg, po qd × 14); *S. typhimurium* A1-R (5 × 10^7^ CFU/100 mL, intravenous (i.v.), per week (qw) × 2); COB (30 mg/kg, 5 mg/kg, po qd × 14) combined with VEM (30 mg/kg, po qd × 14); VEM (30 mg/kg, po qd × 14) combined with *S. typhimurium* A1-R (5 × 10^7^ CFU/100 mL, i.v., qw × 2); rMETase (100 units, intraperitoneal (i.p.), 14 consecutive days, *n* = 10) [9]. For the melanoma tumor from UCSD, the treatment was as follows: 5-fluorouracil (5-FU) (10 mg/kg, i.p., once per week) and CDDP (3 or 5 mg/kg, i.p., once per week) were administered. *S. typhimurium* A1-R (3 or 5 × 10^7^ CFU/body, i.v., once per week) was also injected [34]. Tumor volume (mm^3^) was calculated from length (mm) × width (mm) × width (mm) × 0.5. Data points represent mean ± SD [9]. 

### 3.9. Intratumor l-Methionine Levels

After the completion of rMETase treatment, each tumor was sonicated for 30 s on ice and centrifuged at 12,000 rpm for 10 min. Supernatants were collected and protein levels were measured using the Coomassie Protein Assay Kit (Thermo Scientific, Rockford, IL, USA). l-methionine levels were determined using a high-pressure liquid chromatography (HPLC) procedure we developed previously [81,82]. Methionine levels were normalized to tumor protein by standard procedures. 

## References

[1] Hauschild A., Grob J.J., Demidov L.V., Jouary T., Gutzmer R., Millward M., Rutkowski P., Blank C.U., Miller W.H., Kaempgen E. (2012). Dabrafenib in BRAF-mutated metastatic melanoma: A multicentre, open-label, phase 3 randomised controlled trial. Lancet.

[2] Gandidi S., Massi D., Mandala M. (2016). PD-L1 expression in cancer patients receiving anti PD-1/PD-L1 antibodies: A systemic review and meta-analysis. Crit. Rev. Oncol. Hematol..

[3] Chapman P.B., Einhorn L.H., Meyers M.L., Saxman S., Destro A.N., Panageas K.S., Begg C.B., Agarwala S.S., Schuchter L.M., Ernstoff M.S. (1999). Phase III multicenter randomized trial of the Dartmouth regimen versus dacarbazine in patients with metastatic melanoma. J. Clin. Oncol..

[4] Rabik C.A., Dolan M.E. (2007). Molecular mechanisms of resistance and toxicity associated with platinating agents. Cancer Treat. Rev..

[5] Tang H., Wang Y., Chlewicki L.K., Zhang Y., Guo J., Liang W., Wang J., Wang X., Fu Y.X. (2016). Facilitating T Cell infiltration in tumor microenvironment overcomes resistance to PD-L1 blockade. Cancer Cell.

[6] Larkin J., Ascierto P.A., Dreno B., Atkinson V., Liszkay G., Maio M., Mandal M., Demidov L., Stroyakovskiy D., Thomas L. (2014). Combined vemurafenib and cobimetinib in BRAF-mutated melanoma. N. Engl. J. Med..

[7] McArthur G.A., Chapman P.B., Robert C., Larkin J., Haanen J.B., Dummer R., Ribas A., Hogg D., Hamid O., Ascierto P.A. (2014). Safety and efficacy of vemurafenib in BRAF (V600E) and BRAF(V600K) mutation-positive melanoma (BRIM-3): Extended follow-up of a phase 3, randomised, open-label study. Lancet Oncol..

[8] Sosman J.A., Kim K.B., Schuchter L., Gonzalez R., Pavlick A.C., Weber J.S., McArthur G.A., Hutson T.E., Moschos S.J., Flaherty K.T. (2012). Survival in BRAF V600-mutant advanced melanoma treated with vemurafenib. N. Engl. J. Med..

[9] Kawaguchi K., Igarashi K., Murakami T., Zhao M., Zhang Y., Chmielowski B., Kiyuna T., Nelson S.D., Russell T.A., Dry S.M. (2017). Tumor-targeting *Salmonella typhimurium* A1-R sensitizes melanoma with a BRAF-V600E mutation to vemurafenib in a patient-derived orthotopic xenograft (PDOX) nude mouse model. J. Cell. Biochem..

[10] Slominski A.T., Carlson J.A. (2014). Melanoma resistance: A bright future for academicians and a challenge for patient advocates. Mayo Clin. Proc..

[11] Brożyna A.A., Jóźwicki W., Roszkowski K., Filipiak J., Slominski A.T. (2016). Melanin content in melanoma metastases affects the outcome of radiotherapy. Oncotarget.

[12] Hoffman R.M. (2015). Patient-derived orthotopic xenografts: Better mimic of metastasis than subcutaneous xenografts. Nat. Rev. Cancer.

[13] Fu X., Guadagni F., Hoffman R.M. (1992). A metastatic nude-mouse model of human pancreatic cancer constructed orthotopically with histologically intact patient specimens. Proc. Natl. Acad. Sci. USA.

[14] Hiroshima Y., Maawy A., Zhang Y., Murakami T., Momiyama M., Mori R., Matsuyama R., Katz M.H., Fleming J.B., Chishima T. (2014). Metastatic recurrence in a pancreatic cancer patient derived orthotopic xenograft (PDOX) nude mouse model is inhibited by neoadjuvant chemotherapy in combination with fluorescence-guided surgery with an anti-CA 19–9-conjugated fluorophore. PLoS ONE.

[15] Hiroshima Y., Zhang Y., Murakami T., Maawy A.A., Miwa S., Yamamoto M., Yano S., Sato S., Momiyama M., Mori R. (2014). Efficacy of tumor-targeting *Salmonella typhimurium* A1-R in combination with anti-angiogenesis therapy on a pancreatic cancer patient-derived orthotopic xenograph (PDOX) and cell line mouse models. Oncotarget.

[16] Hiroshima Y., Maawy A.A., Katz M.H., Fleming J.B., Bouvet M., Endo I., Hoffman R.M. (2015). Selective efficacy of zoledronic acid on metastasis in a patient-derived orthotopic xenograph (PDOX) nude-mouse model of human pancreatic cancer. J. Surg. Oncol..

[17] Fu X., Le P., Hoffman R.M. (1993). A metastatic-orthotopic transplant nude-mouse model of human patient breast cancer. Anticancer Res..

[18] Fu X., Hoffman R.M. (1993). Human ovarian carcinoma metastatic models constructed in nude mice by orthotopic transplantation of histologically-intact patient specimens. Anticancer Res..

[19] Wang X., Fu X., Hoffman R.M. (1992). A new patient-like metastatic model of human lung cancer constructed orthotopically with intact tissue via thoracotomy in immunodeficient mice. Int. J. Cancer.

[20] Hiroshima Y., Zhang Y., Zhang M., Maawy A., Mii S., Yamamoto M., Uehara F., Miwa S., Yano S., Murakami T. (2015). Establishment of a patient-derived orthotopic xenograph (PDOX) model of HER-2-positive cervical cancer expressing the clinical metastatic pattern. PLoS ONE.

[21] Fu X.Y., Besterman J.M., Monosov A., Hoffman R.M. (1991). Models of human metastatic colon cancer in nude mice orthotopically constructed by using histologically intact patient specimens. Proc. Natl. Acad. Sci. USA.

[22] Metildi C.A., Kaushal S., Luiken G.A., Talamini M.A., Hoffman R.M., Bouvet M. (2014). Fluorescently-labeled chimeric anti-CEA antibody improves detection and resection of human colon cancer in a patient-derived orthotopic xenograft (PDOX) nude mouse model. J. Surg. Oncol..

[23] Hiroshima Y., Maawy A., Metildi C.A., Zhang Y., Uehara F., Miwa S., Yano S., Sato S., Murakami T., Momiyama M. (2014). Successful fluorescence-guided surgery on human colon cancer patient-derived orthotopic xenograft mouse models using a fluorophore-conjugated anti-CEA antibody and a portable imaging system. J. Laparoendosc. Adv. Surg. Tech..

[24] Furukawa T., Kubota T., Watanabe M., Kitajima M., Fu X., Hoffman R.M. (1993). Orthotopic transplantation of histologically intact clinical specimens of stomach cancer to nude mice: Correlation of metastatic sites in mouse and individual patient donors. Int. J. Cancer.

[25] Hiroshima Y., Zhang Y., Zhang N., Uehara F., Maawy A., Murakami T., Mii S., Yamamoto M., Miwa S., Yano S. (2015). Patient-derived orthotopic xenograft (PDOX) nude mouse model of soft-tissue sarcoma more closely mimics the patient behavior in contrast to the subcutaneous ectopic model. Anticancer Res..

[26] Hiroshima Y., Zhao M., Zhang Y., Zhang N., Maawy A., Murakami T., Mii S., Uehara F., Yamamoto M., Miwa S. (2015). Tumor-targeting *Salmonella typhimurium* A1-R arrests a chemo-resistant patient soft-tissue sarcoma in nude mice. PLoS ONE.

[27] Murakami T., DeLong J., Eilber F.C., Zhao M., Zhang Y., Zhang N., Singh A., Russell T., Deng S., Reynoso J. (2016). Tumor-targeting *Salmonella typhimurium* A1-R in combination with doxorubicin eradicate soft tissue sarcoma in a patient-derived orthotopic xenograft PDOX model. Oncotarget.

[28] Kiyuna T., Murakami T., Tome Y., Kawaguchi K., Igarashi K., Zhang Y., Zhao M., Li Y., Bouvet M., Kanaya F. (2016). High efficacy of tumor-targeting *Salmonella typhimurium* A1-R on a doxorubicin- and dactolisib-resistant follicular dendritic-cell sarcoma in a patient-derived orthotopic xenograft nude mouse model. Oncotarget.

[29] Murakami T., Singh A.S., Kiyuna T., Dry S.M., Li Y., James A.W., Igarashi K., Kawaguchi K., DeLong J.C., Zhang Y. (2016). Effective molecular targeting of CDK4/6 and IGF-1R in a rare FUS-ERG fusion CDKN2A-deletion doxorubicin-resistant Ewing’s sarcoma patient-derived orthotopic xenograft (PDOX) nude-mouse model. Oncotarget.

[30] Hiroshima Y., Maawy A., Sato S., Murakami T., Uehara F., Miwa S., Yano S., Momiyama M., Chishima T., Tanaka K. (2014). Hand-held high-resolution fluorescence imaging system for fluorescence-guided surgery of patient and cell-line pancreatic tumors growing orthotopically in nude mice. J. Surg. Res..

[31] Hiroshima Y., Zhao M., Maawy A., Zhang Y., Katz M.H., Fleming J.B., Uehara F., Miwa S., Yano S., Momiyama M. (2014). Efficacy of *Salmonella typhimurium* A1-R versus chemotherapy on a pancreatic cancer patient-derived orthotopic xenograft (PDOX). J. Cell. Biochem..

[32] Suetsugu A., Katz M., Fleming J., Truty M., Thomas R., Saji S., Moriwaki H., Bouvet M., Hoffman R.M. (2012). Non-invasive fluorescent-protein imaging of orthotopic pancreatic-cancer-patient tumorgraft progression in nude mice. Anticancer Res..

[33] Suetsugu A., Katz M., Fleming J., Truty M., Thomas R., Saji S., Moriwaki H., Bouvet M., Hoffman R.M. (2012). Imageable fluorescent metastasis resulting in transgenic GFP mice orthotopically implanted with human-patient primary pancreatic cancer specimens. Anticancer Res..

[34] Yamamoto M., Zhao M., Hiroshima Y., Zhang Y., Shurell E., Eilber F.C., Bouvet M., Noda M., Hoffman R.M. (2016). Efficacy of tumor-targeting *Salmonella typhimurium* A1-R on a melanoma patient-derived orthotopic xenograft (PDOX) nude-mouse model. PLoS ONE.

[35] Hoffman R.M., Zhao M. (2014). Methods for the development of tumor-targeting bacteria. Expert Opin. Drug Discov..

[36] Zhao M., Yang M., Li X.M., Jiang P., Baranov E., Li S., Xu M., Penman S., Hoffman R.M. (2005). Tumor-targeting bacterial therapy with amino acid auxotrophs of GFP-expressing *Salmonella typhimurium*. Proc. Natl. Acad. Sci. USA.

[37] Zhao M., Geller J., Ma H., Yang M., Penman S., Hoffman R.M. (2007). Monotherapy with a tumor-targeting mutant of *Salmonella typhimurium* cures orthotopic metastatic mouse models of human prostate cancer. Proc. Natl. Acad. Sci. USA.

[38] Zhao M., Yang M., Ma H., Li X., Tan X., Li S., Yang Z., Hoffman R.M. (2006). Targeted therapy with a *Salmonella typhimurium* leucine-arginine auxotroph cures orthotopic human breast tumors in nude mice. Cancer Res..

[39] Zhang Y., Tome Y., Suetsugu A., Zhang L., Zhang N., Hoffman R.M., Zhao M. (2012). Determination of the optimal route of administration of *Salmonella typhimurium* A1-R to target breast cancer in nude mice. Anticancer Res..

[40] Zhang Y., Miwa S., Zhang N., Hoffman R.M., Zhao M. (2015). Tumor-targeting *Salmonella typhimurium* A1-R arrests growth of breast-cancer brain metastasis. Oncotarget.

[41] Uchugonova A., Zhao M., Zhang Y., Weinigel M., König K., Hoffman R.M. (2012). Cancer-cell killing by engineered *Salmonella* imaged by multiphoton tomography in live mice. Anticancer Res..

[42] Liu F., Zhang L., Hoffman R.M., Zhao M. (2010). Vessel destruction by tumor-targeting *Salmonella typhimurium* A1-R is enhanced by high tumor vascularity. Cell Cycle.

[43] Nagakura C., Hayashi K., Zhao M., Yamauchi K., Yamamoto N., Tsuchiya H., Tomita K., Bouvet M., Hoffman R.M. (2009). Efficacy of a genetically-modified *Salmonella typhimurium* in an orthotopic human pancreatic cancer in nude mice. Anticancer Res..

[44] Yam C., Zhao M., Hayashi K., Ma H., Kishimoto H., McElroy M., Bouvet M., Hoffman R.M. (2010). Monotherapy with a tumor-targeting mutant of *S. typhimurium* inhibits liver metastasis in a mouse model of pancreatic cancer. J. Surg. Res..

[45] Hiroshima Y., Zhao M., Zhang Y., Maawy A., Hassanein M.K., Uehara F., Miwa S., Yano S., Momiyama M., Suetsugu A. (2013). Comparison of efficacy of *Salmonella typhimurium* A1-R and chemotherapy on stem-like and non-stem human pancreatic cancer cells. Cell Cycle.

[46] Matsumoto Y., Miwa S., Zhang Y., Hiroshima Y., Yano S., Uehara F., Yamamoto M., Toneri M., Bouvet M., Matsubara H. (2014). Efficacy of tumor-targeting *Salmonella typhimurium* A1-R on nude mouse models of metastatic and disseminated human ovarian cancer. J. Cell. Biochem..

[47] Matsumoto Y., Miwa S., Zhang Y., Zhao M., Yano S., Uehara F., Yamamoto M., Hiroshima Y., Toneri M., Bouvet M. (2015). Intraperitoneal administration of tumor-targeting *Salmonella typhimurium* A1-R inhibits disseminated human ovarian cancer and extends survival in nude mice. Oncotarget.

[48] Yano S., Zhang Y., Zhao M., Hiroshima Y., Miwa S., Uehara F., Kishimoto H., Tazawa H., Bouvet M., Fujiwara T. (2014). Tumor-targeting *Salmonella typhimurium* A1-R decoys quiescent cancer cells to cycle as visualized by FUCCI imaging and become sensitive to chemotherapy. Cell Cycle.

[49] Hiroshima Y., Zhang Y., Zhao M., Zhang N., Murakami T., Maawy A., Mii S., Uehara F., Yamamoto M., Miwa S. (2015). Tumor-targeting *Salmonella typhimurium* A1-R in combination with Trastuzumab eradicates HER-2-positive cervical cancer cells in patient-derived mouse models. PLoS ONE.

[50] Hayashi K., Zhao M., Yamauchi K., Yamamoto N., Tsuchiya H., Tomita K., Hoffman R.M. (2009). Cancer metastasis directly eradicated by targeted therapy with a modified *Salmonella typhimurium*. J. Cell. Biochem..

[51] Hayashi K., Zhao M., Yamauchi K., Yamamoto N., Tsuchiya H., Tomita K., Kishimoto H., Bouvet M., Hoffman R.M. (2009). Systemic targeting of primary bone tumor and lung metastasis of high-grade osteosarcoma in nude mice with a tumor-selective strain of *Salmonella typhimurium*. Cell Cycle.

[52] Miwa S., Zhang Y., Baek K.-E., Uehara F., Yano S., Yamamoto M., Hiroshima Y., Matsumoto Y., Kimura H., Hayashi K. (2014). Inhibition of spontaneous and experimental lung metastasis of soft-tissue sarcoma by tumor-targeting *Salmonella typhimurium* A1-R. Oncotarget.

[53] Kimura H., Zhang L., Zhao M., Hayashi K., Tsuchiya H., Tomita K., Bouvet M., Wessels J., Hoffman R.M. (2010). Targeted therapy of spinal cord glioma with a genetically-modified *Salmonella typhimurium*. Cell Prolif..

[54] Momiyama M., Zhao M., Kimura H., Tran B., Chishima T., Bouvet M., Endo I., Hoffman R.M. (2012). Inhibition and eradication of human glioma with tumor-targeting *Salmonella typhimurium* in an orthotopic nude-mouse model. Cell Cycle.

[55] Kawaguchi K., Murakami T., Chmielowski B., Igarashi K., Kiyuna T., Unno M., Nelson S.D., Russell T.A., Dry S.M., Li Y. (2016). Vemurafenib-resistant BRAF-V600E mutated melanoma is regressed by MEK targeting drug trametinib, but not cobimetinib in a patient-derived orthotopic xenograft (PDOX) mouse model. Oncotarget.

[56] Kawaguchi K., Igarashi K., Murakami T., Chmiewloski B., Kiyuna T., Zhao M., Zhang Y., Singh A., Unno M., Nelson S.D. (2016). Tumor-targeting *Salmonella typhimurium* A1-R combined with Temozolomide regresses malignant melanoma with a BRAF-V600 mutation in a patient-derived orthotopic xenograft (PDOX) model. Oncotarget.

[57] Kawaguchi K., Igarashi K., Chmielowski B., Murakami T., Kiyuna T., Zhao M., Zhang Y., Nelson S.D., Russell T.A., Dry S.M. (2017). *Salmonella typhimurium* A1-R targeting of a chemotherapy resistant BRAF-V600E melanoma in a patient-derived orthotopic xenograft (PDOX) model is enhanced in combination with either vemurafenib or temozlomide. Cell Cycle.

[58] Guo H., Lishko V.K., Herrera H., Groce A., Kubota T., Hoffman R.M. (1993). Therapeutic tumor-specific cell cycle block induced by methionine starvation in vivo. Cancer Res..

[59] Hoffman R.M., Jacobsen S.J. (1980). Reversible growth arrest in simian virus 40-transformed human fibroblasts. Proc. Natl. Acad. Sci. USA.

[60] Kokkinakis D.M., von Wronski M.A., Vuong T.H., Brent T.P., Schold S.C. (1997). Regulation of O6-methylguanine-DNA methyltransferase by methionine in human tumour cells. Br. J. Cancer.

[61] Kokkinakis D.M., Schold S.C., Hori H., Nobori T. (1997). Effect of long-term depletion of plasma methionine on the growth and survival of human brain tumor xenografts in athymic mice. Nutr. Cancer.

[62] Hoffman R.M., Erbe R.W. (1976). High in vivo rates of methionine biosynthesis in transformed human and malignant rat cells auxotrophic for methionine. Proc. Natl. Acad. Sci. USA.

[63] Stern P.H., Mecham J.O., Wallace C.D., Hoffman R.M. (1983). Reduced free-methionine in methionine-dependent SV40-transformed human fibroblasts synthesizing apparently normal amounts of methionine. J. Cell. Physiol..

[64] Stern P.H., Wallace C.D., Hoffman R.M. (1984). Altered methionine metabolism occurs in all members of a set of diverse human tumor cell lines. J. Cell. Physiol..

[65] Stern P.H., Hoffman R.M. (1984). Elevated overall rates of transmethylation in cell lines from diverse human tumors. In Vitro.

[66] Hoffman R.M. (1984). Altered methionine metabolism, DNA methylation and oncogene expression in carcinogenesis: A review and synthesis. Biochim. Biophys. Acta.

[67] Coalson D.W., Mecham J.O., Stern P.H., Hoffman R.M. (1982). Reduced availability of endogenously synthesized methionine for *S*-adenosylmethionine formation in methionine dependent cancer cells. Proc. Natl. Acad. Sci. USA.

[68] Hoffman R.M. (2017). Is DNA methylation the new guardian of genome?. Mol. Cytogenet..

[69] Hoffman R.M. (2017). The wayward methyl group and the cascade to cancer. Cell Cycle.

[70] Lishko V.K., Lishko O.V., Hoffman R.M. (1993). The preparation of endotoxin-free l-methionine-α-deamino-γ-mercaptomethane-lyase (l-methioninase) from Pseudomonas putida. Protein Expr. Purif..

[71] Lishko V.K., Lishko O.V., Hoffman R.M. (1993). Depletion of serum methionine by methioninase in mice. Anticancer Res..

[72] Tan Y., Zavala J., Xu M., Zavala J., Hoffman R.M. (1996). Serum methionine depletion without side effects by methioninase in metastatic breast cancer patients. Anticancer Res..

[73] Tan Y., Zavala J., Han Q., Xu M., Sun X., Tan X., Tan X., Magana R., Geller J., Hoffman R.M. (1997). Recombinant methioninase infusion reduces the biochemical endpoint of serum methionine with minimal toxicity in high-stage cancer patients. Anticancer Res..

[74] Tan Y., Xu M., Tan X., Tan X., Wang X., Saikawa Y., Nagahama T., Sun X., Lenz M., Hoffman R.M. (1997). Overexpression and large-scale production of recombinant l-methionine-α-deamino-γ-mercaptomethane-lyase for novel anticancer therapy. Protein Expr. Purif..

[75] Inoue H., Inagaki K., Sugimoto M., Esaki N., Soda K., Tanaka H. (1995). Structural analysis of the l-methionine γ-lyase gene from Pseudomonas putida. J. Biochem..

[76] Hori H., Takabayashi K., Orvis L., Carson D.A., Nobori T. (1996). Gene cloning and characterization of Pseudomonas putida l-methionine-α-deamino-γ-mercaptomethane-lyase. Cancer Res..

[77] Kawaguchi K., Igarashi K., Li S., Han Q., Tan Y., Kiyuna T., Miyake Y., Murakami T., Chmielowski B., Nelson S.D. (2017). Combination treatment with recombinant methioninase enables temozolomide to arrest a BRAF V600E melanoma growth in a patient-derived orthotopic xenograft. Oncotarget.

[78] Pimiento J.M., Larkin E.M., Smalley K.S., Wiersma G.L., Monks N.R., Fedorenko I.V., Peterson C.A., Nickoloff B.J. (2013). Melanoma genotypes and phenotypes get personal. Lab Investig..

[79] Harris A.L., Joseph R.W., Copland J.A. (2016). Patient-derived tumor xenograft models for melanoma drug discovery. Expert Opin. Drug Discov..

[80] Uchugonova A., Duong J., Zhang N., König K., Hoffman R.M. (2011). The bulge area is the origin of nestin-expressing pluripotent stem cells of the hair follicle. J. Cell. Biochem..

[81] Murakami T., Li S., Han Q., Tan Y., Kiyuna T., Igarashi K., Kawaguchi K., Hwang H.K., Miyaki K., Singh A.S. (2017). Recombinant methioninase effectively targets a Ewing’s sarcoma in a patient-derived orthotopic xenograft (PDOX) nude-mouse model. Oncotarget.

[82] Sun X., Tan Y., Yang Z., Li S., Hoffman R.M. (2005). A rapid HPLC method for the measurement of ultra-low plasma methionine concentrations applicable to methionine depletion therapy. Anticancer Res..

